# The Tumor Suppressor *PTEN* as Molecular Switch Node Regulating Cell Metabolism and Autophagy: Implications in Immune System and Tumor Microenvironment

**DOI:** 10.3390/cells9071725

**Published:** 2020-07-18

**Authors:** Saveria Aquila, Marta Santoro, Annalisa Caputo, Maria Luisa Panno, Vincenzo Pezzi, Francesca De Amicis

**Affiliations:** 1 Department of Pharmacy, Health and Nutritional Sciences; University of Calabria, 87036 Rende, Italy; saveria.aquila@unical.it (S.A.); ms83.santoro@libero.it (M.S.); mamissina@yahoo.it (M.L.P.); vincenzo.pezzi@unical.it (V.P.); 2 Health Center, University of Calabria, 87036 Rende, Italy; 3 Faculty of Medicine and Surgery, Catholic University of the Sacred Heart, 00168 Rome, Italy; annalisa.caputo1995@gmail.com

**Keywords:** Warburg state, cancer metabolism, stroma, immune system

## Abstract

Recent studies conducted over the past 10 years evidence the intriguing role of the tumor suppressor gene Phosphatase and Tensin Homolog deleted on Chromosome 10 *PTEN* in the regulation of cellular energy expenditure, together with its capability to modulate proliferation and survival, thus expanding our knowledge of its physiological functions. Transgenic *PTEN* mice models are resistant to oncogenic transformation, present decreased adiposity and reduced cellular glucose and glutamine uptake, together with increased mitochondrial oxidative phosphorylation. These acquisitions led to a novel understanding regarding the role of *PTEN* to counteract cancer cell metabolic reprogramming. Particularly, *PTEN* drives an “anti-Warburg state” in which less glucose is taken up, but it is more efficiently directed to the mitochondrial Krebs cycle. The maintenance of cellular homeostasis together with reduction of metabolic stress are controlled by specific pathways among which autophagy, a catabolic process strictly governed by mTOR and *PTEN*. Besides, a role of *PTEN* in metabolic reprogramming and tumor/stroma interactions in cancer models, has recently been established. The genetic inactivation of *PTEN* in stromal fibroblasts of mouse mammary glands, accelerates breast cancer initiation and progression. This review will discuss our novel understanding in the molecular connection between cell metabolism and autophagy by *PTEN*, highlighting novel implications regarding tumor/stroma/immune system interplay. The newly discovered action of *PTEN* opens innovative avenues for investigations relevant to counteract cancer development and progression.

## 1. Introduction

The tumor suppressor gene Phosphatase and Tensin Homolog deleted on Chromosome 10 (*PTEN*) is often mutated in human tumors with germline mutations causing cancer-predisposition syndromes [1]. Genetic inactivation of *PTEN* is frequently found in glioblastomas, melanomas, endometrial, prostate, colon, and bladder cancers, and reduced *PTEN* expression has also been observed in lung and breast cancer [2]. Loss of *PTEN* function can occur by mutations or deletions, epigenetic silencing, transcriptional repression or by micro RNA (miRNA) regulation [3].

*PTEN* is a protein–phosphatase and a lipid–phosphatase. As a lipid–phosphatase, *PTEN* decreases the cellular amount of phosphatidylinositol-3,4,5-phosphate (PIP3) which is an important second messenger mediating different signaling pathways [4]. Inactivation of *PTEN* enzymatic activity leads to induction of cell proliferation and inhibition of cell death, causing cancer development and progression [5]. Several studies carried out in both human samples and hypomorphic *PTEN* mice indicate a continuum model of *PTEN* tumor suppression, rather than a stepwise alteration of *PTEN* levels [6]. Indeed, even partial loss of *PTEN* function is sufficient to promote some cancer types and a reduction in *PTEN* levels below 50% further accelerates cancer progression [7]. Notably, studies carried out *in vitro* and in *PTEN* mouse models (see Table 1) show that even subtle reductions in *PTEN* enzymatic activity influence cancer susceptibility, demonstrating the existence of *PTEN* tumor suppressor pathways [5]. Nevertheless, it is reported that complete loss of *PTEN*, resulting in a tumor suppressor p53 increase can counteract tumor growth by activating an effective fail-safe mechanism—cellular senescence—in the prostate cells [8]. The above described effect has important relevance, since it indicates that both genes need to be ablated for prostate cancer progression.

New insights into molecular mechanisms of cancer demonstrate dysregulation of cellular metabolism, increase of glucose uptake and fermentation of glucose to lactate. In this concern, a metabolic point of view of *PTEN* function emerges: *PTEN* inactivation produces fatty acid accumulation which leads to non-alcoholic fatty liver disease and long-latent liver tumorigenesis [9].

Moreover, it is accepted that mechanisms for *PTEN* dimerization and inactivation could be deregulated in cancer [10]. *PTEN* is secreted into the extracellular environment for uptake by recipient cells, thus also working as a tumor suppressor in a cell non-autonomous manner [11].

Interestingly, a role of *PTEN* in tumor/stroma interactions in cancer models is increasingly supported [12]. Genetic inactivation of *PTEN* in stromal fibroblasts of mouse mammary glands accelerates breast cancer initiation and progression. Specifically, the tumor suppressor activity of *PTEN* in the stroma is mediated by the regulation of multiple signaling pathways, such as Ras proto-oncogenes, Protein kinase B (PKB), also known as AKT and c-Jun *N*-terminal kinase (Jnk) networks, which modulate the transcription factor Ets2 and was able to reduce tumor growth and progression [13].

The above reported findings delineate a complex scenario of tumor suppressor function of *PTEN*. The classical role of *PTEN* in human disease is played through the modulation of the phosphoinositide 3-kinase (PI3K) activity. Indeed *PTEN*, dimethylated at arginine 159 (R159) is mutated at R159 in cancers, losing the capability to inhibit the PI3K–AKT pathway targeting the mammalian target of rapamycin (mTOR) [14,15,16]. The role of mTOR is critical for sensing the nutritional status of the cell, regulating initiation of autophagy and the energy expenditure [17]. Cells reprogram their metabolism very early during carcinogenesis, and this event is critical for the establishment of other cancer hallmarks. Thus, *PTEN* is emerging as a crucial sorter of a metabolic network, controlling specific gene expression and pathways. These new findings suggest an intriguing point of view of *PTEN* biology and function.

Here, we will outline *PTEN* as an essential determinant of a tumor suppressor metabolic state influencing the complex interplay between the tumor and immune system. First, the biochemical functions of *PTEN* on cell metabolism and autophagy will be discussed. Then, the role of *PTEN* in tumor microenvironment remodeling will be underlined. The recent advances in our understanding of *PTEN* biological roles may help to identify new opportunities to improve *PTEN* function for cancer therapy.

## 2. Biochemical Functions of *PTEN* and Cancer Metabolism

*PTEN* as lipid–phosphatase [27,28] acts as negative regulator of the class I phosphatidylinositol 3-kinases (PI3Ks) which phosphorylates phosphatidylinositol-4,5-bisphosphate (PIP2) to generate the second messenger phosphatidylinositol-3,4,5-trisphosphate (PIP3). The PIP3 induces molecular signaling, such as the activation of AKT kinases, which act on molecular targets relevant for different biological roles, like regulation of cell growth, cell proliferation, vesicle trafficking, angiogenesis, anabolic metabolism and cancer [29]. Thus, *PTEN* is relevant for the control of the nutrient-responsive signaling involved in protein synthesis and transcription [30].

### 2.1. PTEN Intercepts AKT-Dependent Metabolic Pathways

**Figure 1:**
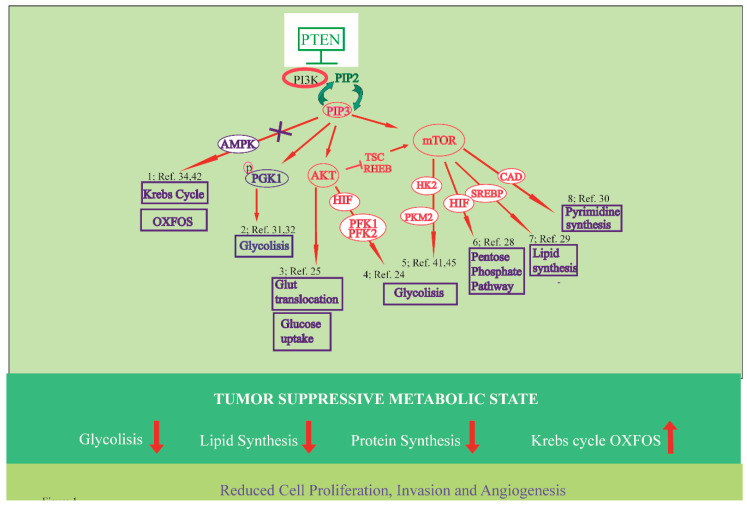
A schematic summary of metabolic pathways (upper panel) determining the tumor suppressive activities (lower panel) for *PTEN*. See the text for details. (1) *PTEN* through AMPK induces Krebs cycle and OXFOS. (2) *PTEN* suppresses glycolysis through PGK1 inhibition. (3) *PTEN* blocks AKT induced Glut1 translocation and glucose uptake. (4) *PTEN* blocks mTOR-induced HIF1, PFK1, and glycolysis. (5) *PTEN* blocks mTOR-induced PKM2 transcription. (6) *PTEN* decreases PPP flux. (7) *PTEN* blocks mTOR-induced SREBP transcription factor and lipid synthesis. (8) *PTEN* reduces pyrimidine synthesis.

Activated AKT is a central regulator of oncogenic metabolism. It is accepted that AKT stimulation pushes the glycolytic metabolism of tumor cells [31,32]. The activation of AKT, resulting from *PTEN* loss, stabilizes the enzyme phosphofructokinase-1 (PFK1) [33] (see Figure 1, point 4) thus promoting glycolysis, cellular proliferation, and brain tumor growth. Specific phosphorylation of AKT rises cellular glucose uptake and is crucial to induce glucose transporter 1 (Glut1) or Glut4 translocation to the plasma membrane of adipocytes (see Figure 1, point 3) [34]. In addition, AKT stimulates the mTOR, a serine/threonine kinase that is part of two distinct complexes, mTOR Complex (TORC) 1, and TORC2 which directly control cell metabolism and growth in response to environmental signals [35].

### 2.2. PTEN Intercepts mTOR Dependent Metabolic Pathways

The mTOR pathway is one of the most deregulated signaling pathways in human cancer, and constitutive activation of mTORC1 is very frequently observed in different tumors [36]. Indeed, mTOR acts on many anabolic pathways sustaining cell proliferation, such as glycolysis and the pentose phosphate pathway (PPP), through regulation of hypoxia-inducible factor (HIF)1 (see Figure 1, points 5, 6) [37]. It also stimulates lipid synthesis by activating the transcription factor sterol regulatory element-binding protein (SREBP)1 [38] (see Figure 1, point 7) and nucleotide through regulation of the PPP and by activation of an enzyme of pyrimidine synthesis (see Figure 1 point 8) [39].

The PI3K–AKT–mTOR pathways play a crucial role in cancer development through an elevated number of components within the cascade, whose level or activity is found altered, and recent studies suggest that tumor suppressive activities for *PTEN* are exerted at biochemical metabolic pathways. The phosphatase action of *PTEN* regulates phosphoglycerate kinase 1 (PGK1) glycolytic enzyme (see Figure 1, point 2) capable of autophosphorylation. Loss of *PTEN* function in cancer cells causes augmented PGK1 autophosphorylation, glycolysis, and ATP production, induction of cancer cell proliferation, and tumorigenesis. [40,41]. Thus, *PTEN* exerts multiple functions that occur in different cellular compartments for which the phosphatase domain is required to inhibit cancer development [18,42]. Consequently, *PTEN* may represent one of the main targets for cancer therapy.

## 3. The Multifaceted Action of *PTEN* on Cell Metabolism

Cancer cells reprogram their metabolism to sustain abnormal cell proliferation, survival, and long-term maintenance [43]. The common feature of this altered metabolism is increased glucose uptake and fermentation of glucose to lactate. This phenomenon is observed even in the presence of completely functioning mitochondria and is known as the Warburg effect, the most important metabolic hallmark of cancer cells [44] which prefer the glycolysis pathway even in the presence of normal or high oxygen tension. Although aerobic glycolysis is an inefficient mean of generating ATP compared to the amount obtained by mitochondrial respiration, cancer cells adopt the metabolic reprogramming as approach for energy compensation [45]. Indeed, the rate of glucose metabolism through aerobic glycolysis is higher such that the production of lactate from glucose occurs 10–100 times faster, compared with the complete oxidation of glucose in the mitochondria. In cancer cells, the amount of ATP synthesized over any given period of time is comparable when either form of glucose metabolism is utilized [46]. In this concern, microarray analysis shows that genes of the glycolysis pathway are overexpressed in the majority of clinically relevant cancers. Besides, the upregulation of plasma membrane glucose transporters and changes in key enzymes involved in glucose utilization have been observed in many tumor types [46] likely contributes to the avid uptake of glucose, even when its availability is becoming insufficient, because of the continuous growth of the tumor. Some tumors show increased expression and activity levels of hexokinase (HK) isoforms, PFK1, PFK2, aldolase (ADO), phosphoglycerate kinase (PGK), enolase (ENO), and pyruvate kinase (PK) [47] which increase pyruvate production from glucose breakdown. Inhibition of glycolysis in cancer cells is considered an alternative therapeutic strategy for cancer patients; thus, drugs targeting the abovementioned controlling enzymes in tumor glycolysis could have new promising applications.

### 3.1. Regulation of Glucose Metabolism

Multiple oncogenic pathways, such as Ras-dependent, Myc or PI3K, favor glycolysis over oxidative phosphorylation, while many tumor suppressors such as p53, Von Hippel–Lindau (VHL), or liver kinase B1 (LKB1) negate the “Warburg effect” [48,49]. Accordingly, in vivo models with tumor suppressor *PTEN* expression elevated to varying levels (*PTEN* tg mice) have revealed mouse embryonic fibroblast (MEF) metabolic changes in which less glucose is taken up, but it is more efficiently directed to the mitochondrial Krebs cycle thus consistent with an “anti-Warburg effect” (see Figure 1, point 1). Specifically, *PTEN* tg MEFs present higher levels of peroxisome proliferator-activated receptor gamma coactivator 1-alpha (PGC1α), a transcriptional coactivator which regulates mitochondrial biogenesis and energy metabolism [42,50]. These mice exhibit increased oxygen consumption and energy expenditure [51]. Moreover, MEFs show an augmented number of mitochondria, together with ATP production and oxygen consumption and lower levels of lactate secretion. All these features indicate that *PTEN* decreases the glycolytic rate and favors oxidative phosphorylation. Thus, in summary, *PTEN* tg mice exhibit an unexpected cancer-resistant and very unique metabolic state which is the outcome of the ability of *PTEN* to regulate metabolism at multiple levels both from the cytosol and from the nucleus.

Glucose consumption is limted by *PTEN* in cancer cells, by preventing the expression of Glut1 on plasma membrane. At a molecular level, *PTEN* blocks AKT activation which controls the localization of Glut1 in the plasma membrane [46]. The regulator of glycolysis, HK2, is increased by the combined loss of *PTEN* and p53. The mechanism of *PTEN* deletion is dependent on activation of the AKT–mTORC1 and HK2 protein synthesis. In prostate cancer models with *PTEN*/p53-deficiency, aerobic glycolysis dependent on HK2 drives tumor growth [52]. Very recently, it has been reported that the regulation of *PTEN*/AKT/HK2 could be targeted to overcome cancer resistance to cisplatin treatment [53]. Moreover, *PTEN* reduces the levels of pyruvate kinase muscle isozyme (PKM) 2 which catalyzes the last step of glycolysis (see Figure 1, point 5) and its expression is associated to the “Warburg effect” of cancer cells [49]. The transcription of PKM2 is induced by mTOR and lower levels of PKM2 are found in *PTEN* transgenic cells [42]. Remarkably, *PTEN* counteracts the glyoxalase dependent PI3K/AKT/mTOR/p-PKM2(Y105)-axis inducing an elevated glycolytic rate and cell proliferation in prostate cancer.

Loss of *PTEN*, via suppressive effects on anaphase-promoting complex (APC) and its coactivator Cdh1-mediated ubiquitination, could stabilize 6-phosphofructo-1-kinase/fructose-2,6-biphosphatase isoform 3 (PFKFB3) family member [54] which is critical for the first commitment step of glycolysis and whose activity has been implicated in cancer [44].

### 3.2. Regulation of Glutamine Metabolism

Glutaminolys, as well as glycolysis, is another mainstay for energy production and anabolism in cancer cells. A recent elegant study demonstrates that *PTEN* loss brings a hyperglycolytic phenotype which would render T-cell acute lymphoblastic leukemia (T-ALL) cells resistant to Notch signaling pathway inhibition [55], given the aberrant activation of Notch in over 60% of T-ALL cases. Besides, the same T-cells are less sensitive to inhibition of glutaminolysis as result of increased glucose-derived carbon input to the Krebs cycle. Notably, *PTEN* can affect glutaminolysis as a crucial point in metabolic reprogramming, influenced by Notch. Glutaminases (GLS1 and GLS2) produce glutamate from glutamine and activate step one in the glutaminolytic pathway. Glutamine consumption is reduced by *PTEN* s due to the concomitant degradation of GLS1 [50] which is pro-oncogenic [56]; however, GLS2 is anti-oncogenic [57]. The oncogene Myc upregulates only GLS1 while the onco-suppressor p53 stimulates GLS2 [58]. In agreement with these data, *PTEN* inhibits the glutaminase GLS1, further supporting the tumor-suppressive activity of *PTEN* in cancer metabolism.

Notably, studies on the effects of suppression of *PTEN* expression by a specific miRNA, such as miR-181a, evidence increased AKT phosphorylation and lactate production, causing cell proliferation [59]. Specifically, miR-181a via *PTEN* is a crucial determinant of metabolic reprogramming in colon cancer, while no significant changes in the critical components of mTORC2 are observed.

### 3.3. Regulation of Krebs Cycle and Oxidative Phosphorylation

The Krebs cycle occupies a central position in metabolism and meets most of cell energy requirement by the complete oxidation of acetyl-CoA, a key product in the catabolism of carbohydrates, fatty acids and amino acids, to CO_2_. Recently in non-transformed thyrocytes of a *PTEN*-deficient mouse model, the constitutive *PTEN* loss affects Krebs cycle and oxidative phosphorylation, with defective mitochondria and compensatory metabolic switch to glycolysis [18]. Furthermore, impairment of the Krebs cycle is associated to pathological conditions including cancer, whereas genetic and epigenetic alterations of Krebs cycle enzymes favor the shift of cancer cells from oxidative phosphorylation to anaerobic glycolysis. Conversely, recent data from transgenic mice models carrying additional copies of *PTEN* (*PTEN* tg) indicate that elevation of this gene induces a tumor suppressive metabolic state [42]. The *PTEN* tg mice resulted in homogeneous and systemic *PTEN* overexpression (2–3 fold higher than normal mice) and shared remarkably overlapping phenotypes. The elevation of *PTEN* results in healthy metabolism characterized by increased energy expenditure and reduced body fat accumulation. Cells derived from these mice are resistant to oncogenic transformation and show reduced glucose and glutamine uptake and increased mitochondrial oxidative phosphorylation. These results demonstrate that *PTEN* is a crucial node for the control of tumorigenesis related to dysregulated cell metabolism (see Figure 1, point 1).

Notably, *PTEN* plays a key role in insulin-mediated oxidative stress and genomic damage in a human hepatocyte cell line and in vivo models. Increased reactive oxygen species (ROS), stress-proteins, and genomic damage in the liver of *PTEN* haplo-deficient mice maintained with a high fat diet, is reported [19] further supporting a causative role of *PTEN* in hepatic and extrahepatic carcinogenesis observed in obese subjects. Surprisingly, in *PTEN* tg mice, oxidative phosphorylation is augmented together with the ROS amount [42]. Since *PTEN* overexpression is associated with cancer protection, this increase in ROS levels is not sufficient to exert relevant effects on DNA. Besides, it should be evidenced that *PTEN* induces the transcription of genes mediating antioxidant activity through the Forkhead box O (FOXO)3 transcription factors [60].

## 4. *PTEN*, Autophagy, and Cancer

Recent acquisitions demonstrate that cell metabolism is tightly connected with autophagic pathways [61,62]. Specific enzymes such as AMP-activated protein kinase (AMPK), protein kinase A (PK) A, and mTOR play a role in cellular energy homeostasis and control autophagy process together with respiration amending energy requests for cellular behavior. Cell proliferation is possible in particular energy conditions influenced by cellular ATP demands. Autophagy, which is a catabolic process, produces glucogenic and ketogenic amino acids and is enabled to fuel the Krebs cycle at multiple entry points thus contributing to the ATP supply. During autophagy, lysosomes degrade damaged cell components thus precursor molecules, energy for neo-synthesis, and metabolic requests are generated. Consequently, autophagy retains an adaptive response through which cells tolerate unfavorable conditions. Moreover, autophagy is a protective mechanism able to avoid hazardous situations in the cell (e.g., increasing ROS or DNA damage), preventing cancer initiation and progression.

Interestingly, genetic manipulation causing impaired autophagy in mice demonstrates that tumor formation is prevented by autophagy process. For instance, mice with allelic loss of BECLIN 1, the master autophagic gene, show augmented susceptibility to tumor development compared to wild-type mice [63]. Although autophagy sustains tumor metabolism and growth during Ras-induced transformation and tumorigenesis [64], compelling evidence suggests that onco-suppressors mediate autophagy process [63] particularly targeting mTOR. Specifically, AMPK, LKB1, ttuberous sclerosis proteins (TSC) ½, and *PTEN* induce autophagy, equally, oncogenes that activate mTOR block autophagy.

### 4.1. Interaction between PTEN Signaling and Autophagic Alterations

In glioblastomas *PTEN* is frequently mutated s and ectopic expression of functional *PTEN* in U87MG glioma cells, induces the autophagic flux and the lysosomal mass. However, proteasome activity and protein ubiquitination are inhibited. Interestingly, the effects are independent of *PTEN* lipid phosphatase activity on the PI3K/AKT/mTOR signaling pathway [20]. These results propose a novel mTOR-independent signaling pathway by which *PTEN* can act on intracellular protein degradation influencing autophagy. The molecular components by which the tumor suppressor *PTEN* regulates proteolytic systems related to cancer development could represent innovative therapeutic targets for patient therapy.

The oncogene *RAS* and p53 loss are crucial determinants of pancreatic cancer, and loss of autophagy contributes to the progression of the disease [65]. Besides, animals with deletion of the key modulator Autophagy related (Atg) 7 and hemizygous for *PTEN*, develop pancreatic ductal adenocarcinoma [66]. Indeed, blocking of autophagy together with *PTEN* hemizygosity permit tumor development and an early death related to pancreatic cancer matched to autophagy-competent mice. Specifically, autophagy-deficient tumors are also *PTEN*-deficient but notably wild-type for p53, further strengthening the crucial protective role of *PTEN*.

Published findings show that *PTEN* can determine autophagy’s contribution to tumor development by DNA damage, increased inflammation, metabolic reprogramming, and oxidative stress increase. For instance, molecular mechanisms controlling autophagy are influenced by the cellular messenger nitric oxide (NO) formed by distinct isoforms of nitric oxide synthase (NOS). Specifically, NOS1 stimulates the survival of nasopharyngeal carcinoma cells through S-nitrosylation of *PTEN* proteins, induction of AKT/mTOR, and block of the autophagic flux [67].

Generally, autophagy effects are context dependent to exert a tumor suppressive or oncogenic action; however, a very elegant study showed an association of a casein kinase 1 alpha 1 (CK1α)-dependent autophagic mechanism and the tumor-suppressor role exerted by *PTEN*/Atg7 signal in xenograft models as well as in lung cancers. [68]. Specifically, CK1α increased *PTEN* stability and activity counteracting *PTEN* polyubiquitination and abrogating *PTEN* phosphorylation. These events account for AKT inhibition and FOXO3a-induced transcription of Atg7. The effects of *PTEN* deficiency are investigated also in hepatocyte of *PTEN*-deficient mice. The authors describe a reduction of autophagosomes formation and maturation, inhibition of Atg conjugation reactions, and induction of insulin pathway, suggesting that hepatic *PTEN* loss causes relevant action on the whole mice metabolism [21]. However further characterization of Atg proteins is necessary to improve the comprehension of autophagy defects in the *PTEN* loss and cancer.

### 4.2. Regulation of Autophagy and Cell Growth by PTEN in Endocrine Related Cancer

**Figure 2:**
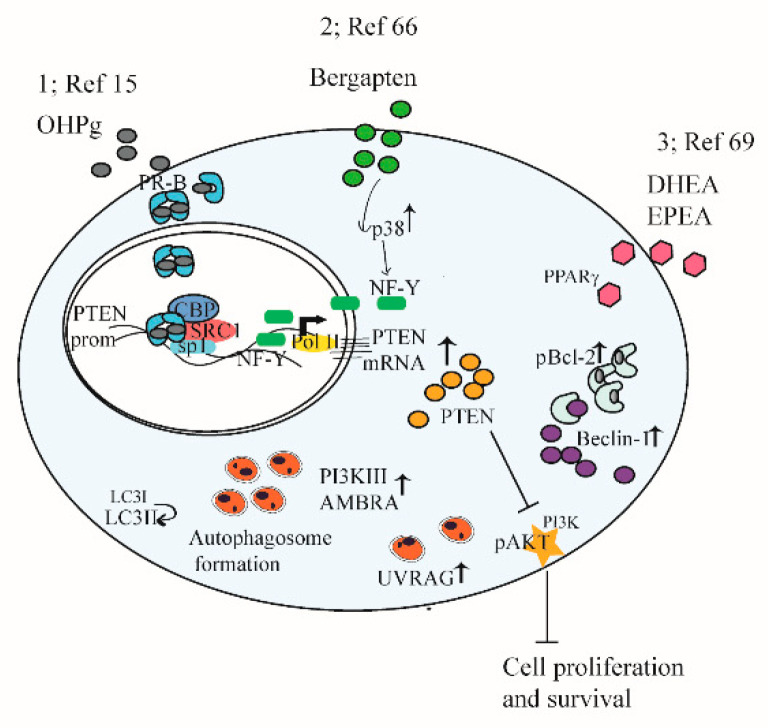
A schematic summary illustrating mechanisms targeting *PTEN* and autophagy in cancer cells. See the text for details. (1) The functional interplay between progesterone receptor-B and *PTEN*, via AKT, modulates autophagy in breast cancer cells. (2) *PTEN* as a key target of Berga*PTEN* action in breast cancer cells for the induction of autophagy. (3) DHEA and EPEA through PPARγ-increased expression *PTEN*, resulting in the inhibition of AKT–mTOR pathways, induction of Bcl-2 phosphorylation, its dissociation from Beclin-1, and autophagy induction.

Given the abovementioned phenotypes associated with increased *PTEN* dosage, some studies investigate the role of *PTEN* through steroid receptors in endocrine-related cancer [15,69]. In estrogen receptor (ER) +/progesterone receptor (PR) + breast cancer cells, a novel functional connection between *PTEN* and autophagy is described as a tumor suppressor pathway (see Figure 2, point 1). We show that progesterone (OHPg)/PR-B induce a genomic mechanism involving *PTEN* gene transcription. The *PTEN* gene is located on the human chromosome 10 q23.3, consisting of nine exons. The 5′-UTR sequence contains a strong promoter mapped to the region between −551 and −220 bases upstream of the translation start codon [70] PR-B recruits at an Sp1-rich region within the *PTEN* gene promoter. Increase of *PTEN* levels reduces PI3K/AKT signals, switching on the autophagic flux by enhancing the expression of the regulator of membrane trafficking in autophagy (UVRAG) driving a reduction of breast cancer cell proliferation [15].

Additional studies address how induction of *PTEN* expression causes autophagy in hormone-dependent breast cancer cells. For instance, the psoralen bergapten reveals anti-survival effects by inducing *PTEN* through p38 mitogen-activated protein kinase (p38MAPK), nuclear transcription factor Y (NF-Y) axis, and autophagy [71]. Indeed, autophagy regulators, such as BECLIN 1, Class III PI 3-kinase, UVRAG, and aativating molecule in Beclin 1-regulated autophagy (AMBRA) 1 protein expression increase, together with conversion of the microtubule-associated protein 1A/1B-light chain 3 (LC3) I to LC3-II. Moreover, autophagic vesicles are evidenced in treated cells. The autophagy process is crucially related to the increase of *PTEN* as demonstrated by specific siRNA studies (see Figure 2, point 2).

In agreement with these results, a functional molecular crosstalk between steroid receptors, *PTEN* and autophagy is demonstrated in testicular germ cells. In TCAM2 cell line, estradiol through ERβ increases *PTEN* gene expression and promoter activity. Both *ERβ* and *PTEN* are responsible for the reduction of cell survival, suggesting a molecular liaison, pAKT is inhibited, and autophagy-related markers increase, indicating the autophagy induction by estradiol [72]. Through DNA fragmentation, cleavages of caspase 9 and Poly (ADP-ribose) polymerase (PARP1), involved in DNA repair processes, are not evidenced; thus, authors [72] propose that necroptosis and/or parthanatos occur, instead of apoptosis. These data demonstrate that *ERβ*/*PTEN* play a protective action in testicular germ cells.

In prostate cancer, *PTEN* deletions are associated with abnormal induction of AKT–mTOR and androgen receptor (AR) signaling pathways. A recent study evidenced that conditional homozygous deletion of histone deacetylase (HDAC) 3, AKT inhibition, and AR reduction in *PTEN*-deficient mouse models suppresses prostate tumorigenesis and progression [73], suggesting in the HDAC 3 inhibition together with *PTEN* inducers new possible therapeutic approaches for prostate cancer patients.

Protein levels of *PTEN* are induced by activation of peroxisome proliferator-activated receptor gamma (PPARγ) which mediates the interplay between cancer and metabolic syndromes, diabetes, and obesity. The studies signify the importance of PPARγ and *PTEN*’s interaction in cancer prevention [74]. In ER+ PR+ breast cancer cells, dietary omega-3 long-chain polyunsaturated fatty acids, docosahexaenoic acid (DHEA), and eicosapentaenoic acid (EPEA) enhance *PTEN* via PPARγ, blocking AKT–mTOR pathways. Moreover, DHEA and EPEA stimuli increase B-cell lymphoma 2 (Bcl-2)-phosphorylation causing its dissociation from BECLIN 1 which results in autophagy induction [75]. In summary (see Figure 2, point 3), data show the anti-proliferative action of two omega-3 ethanolamides involving *PTEN*-mediated induction of autophagy, suggesting their potential use as breast cancer preventive and/or therapeutic agents.

### 4.3. Regulation of EMT and Invasion through Autophagy by PTEN

Recent findings in cancer progression investigate new possible molecular mechanisms regulating invasion, epithelial mesenchymal transition (EMT) [76,77], and the possible link with autophagy. It is reported that (synaptojanin 2 binding protein) SYNJ2BP, crucially involved in the pathogenesis of metastatic breast tumors, is responsible of the degradation of *PTEN* through the lysosomes; particularly, SYNJ2BP stimulates the recruitment of *PTEN* at autophagy-lysosomes, the autophagosome cargo (p62) expression, and LC3-I to LC3-II conversion. Collectively, these data evidence that SYNJ2BP causes the autophagic degradation of *PTEN* which is related to the activation of Snail, a zinc-finger transcriptional repressor controlling EMT thus promoting both EMT and invasion in breast cancer models [78]. Further study investigates the effects of *PTEN*, lncRNA growth arrest-specific 5 (GAS5), its target genes, and microRNA-222-3p (miR-222-3p) on motility, invasive capacity, and autophagic flux in colorectal cancer cells (CRCs). The results indicate, with elegant experimental design, that lncRNA GAS5 exerts an anti-oncogenic action in CRCs counteracting the inhibitory effects of miR-222-3p on *PTEN* [79] which acquires novel relevance for patient’s treatment.

## 5. *PTEN*, Immune System, and Cancer

Through autophagy *PTEN* determines the fate of the tumor [61]. In addition, the biology of the immune system dictates tumor initiation and progression [80] through the balance between effector and tolerogenic response modulated by autophagy [81]. Autophagy influences different biological functions of different cell types of the immune system such as natural killer cells, dendritic cells, macrophages, and T and B lymphocytes. It could modulate the secretion of cytokines and antibodies which also have effects on the autophagic process itself. Transforming growth factor -β, interferon-γ, and several interleukins (IL) are stimulators, while IL-4, IL-10, and IL-13 counteract autophagy [82]. Autophagy can be stimulated by the activity of innate immune receptors, such as Toll-like receptors [83], and in adaptive immunity, it is determinant for antigen presentation, lymphocyte differentiation, and cytokines secretion with onco-suppressor activity [84]. Therefore, an ideal therapeutic portfolio could be integrated by autophagy-based inducers, namely, *PTEN* inducers together with existing therapeutic strategies to elicit cancer cell death and patients’ responses.

Unfortunately, malignant tumors can avoid immune surveillance through the expression of components sustaining immune tolerance. One example is the programmed cell death-ligand-1 (PD-L1) secreted by cancer cells, which interacts with specific receptor, such as programmed cell death-1 (PD-1), expressed by lymphoid and non-lymphoid immune cells. The interaction between PD-L1 and PD-1 causes reduction of lymphocyte response [80]. Indeed, interference of PD-L1/PD-1 axis by blocking molecules, produces durable responses in numerous cancer types, such as advanced melanoma, bladder cancer, kidney cancer, and glioblastoma [85]. However, often the therapy fails and patients relapse. In this concern autophagy inducers could improve the action of immunotherapy by warranting an ideal secretion of immune-stimulatory factors, providing antigens to the immune cells and eventually modulating immune responses for tumor recognition and rejection [86]. Conversely, an autophagic process, can hinder the immune responses thus decreasing immunotherapy effects. Although the molecular basis of immune resistance is still to be defined, a number of studies demonstrate the role played by *PTEN*.

### 5.1. Role of PTEN in T Cells Function

Loss of *PTEN* crucially contributes to immune resistance in cancer disease. For instance, in cancer cells and in mouse models of melanoma, *PTEN* knockout counteracts T cells’ action on tumor cells and reduces T cell trafficking into cancer tissue [22]. Loss of *PTEN* induces a mechanism regulated by immunosuppressive cytokines, determining a reduction of T cells’ infiltration into tumor tissue and inhibition of autophagy and consequently T cell–mediated cell death. In patients, *PTEN* loss correlates with inferior outcomes with PD-1 inhibitor therapy. The use of selective PI3Kβ inhibitor ameliorates the efficacy of immunotherapy in murine models [22].

In humans, cancer development is frequently associated with chronic immunosuppression and inflammatory diseases. For instance, head and neck squamous cancers are characterized by neutrophils in inflammatory infiltrates. Neutrophils found in mouse tumors antagonize effector T cell function, support the generation of immunosuppressive T cell populations, and inhibit the lysis of tumor cells by cytotoxic T cells or natural killer (NK) cells [87]. In mouse, biallelic inactivation of LKB1, which is involved in starvation-induced autophagy, and *PTEN* (so called LP mice), causes lung squamous cell carcinoma, exhibiting histologic pattern and gene expression showed in human disease. These tumors are characterized by tumor-associated neutrophils and by epithelial cell populations expressing high levels of the CXC chemokines which regulate the motility and adhesion of neutrophils. Moreover, cancer stem cells population show enhanced tumor-propagating capability as well as elevated expression of the immune evasion marker (PD-L1) indicating that these cells display immune evasion capacity [88]. The results also indicate the inefficacy of checkpoint inhibitors in these models, suggesting that *PTEN* deletion could be retained, a strong indicator of non-responder tumors. Notably, a meta-analysis of the data of non-small-cell lung cancer (NSCLC) patients. treated by immunotherapy showed that while p53, epidermal growth factor receptor (EGFR), and LKB mutations are not correlated with the immune response, *PTEN* was associated with resistance to anti-PD-1 therapy [89].

Interestingly, further studies established that PD-1 may be retained an haploinsufficient tumor suppressor in T cell lymphoma. The molecular mechanism appears to be related to PD-1 activity which increases *PTEN* levels and reduces AKT and protein kinase C signals in pre-malignant cells. Therefore, reinforcing *PTEN* activity could be a potential strategy for treatment of these types of cancers; conversely, checkpoint inhibitors could reactivate T cell in limphoma patients [90].

Systemic immunosuppression was evidenced in patients with glioblastoma and in glioma animal models [91]. Specifically, glioblastomas had a reduced amount of infiltrating T cells and harbored a quite small number of somatic mutations in respect to other tumors. However, a significant augmentation of *PTEN* mutations, correlated with immunosuppressive expression profile in glioblastomas patients resistant to anti-PD-1 immunotherapy [91]. Meanwhile, a further study in glioblastoma shows that expression of the PD-L1 increases in human glioma in the case of *PTEN* deletion as well as dysregulated PI3K signal [92]. In summary, the effects of specific drugs need further investigations, although, in most cases, T cells’ functions might be improved by employing specific agents, such as *PTEN* inducers or PI3K inhibitors, in different cancer patients.

### 5.2. Role of PTEN in Macrophages Function

Similarly, immune response involves macrophages. These cell types after pathogens and other noxious stimuli, become activated and initiate immune responses. The different phases of macrophage activation are defined as M1 and M2 polarization, characterized by specific phenotypes induced by inflammatory stimuli and influenced by the cellular context. Macrophages isolated from metastatic human cancers usually present an M2-like phenotype, consistent with the cancer-related inflammation. The PI3K/AKT pathway and its downstream targets play crucial role in the activated macrophages consequently *PTEN*, acting on various converging pathways, controls macrophage biology. It is reported that *PTEN* regulates macrophages activation by increase of Arginase I release [93] leading to a hypoinflammatory environment. Further evidences demonstrate that a potent inhibitor of *PTEN*, VO-OHpic, inhibits adverse cardiac remodeling due to the macrophages polarization. Pro-inflammatory M1 macrophages are reduced while anti-inflammatory M2 macrophages are increased in animal models treated with doxorubicin which unfortunately can stimulate cardiomyopathy [94].

## 6. *PTEN* and Tumor Microenvironment

Tumor growth and metastatic process rely on intrinsic characteristics, the response of host tissue and signals coming from tumor microenvironment (TME). The TME consists of blood vessels close to cancer cells, the extracellular matrix (ECM), and other non-cancer cells [95]. These stromal cells comprise fibroblasts (CAFs), immune cells such as T and B lymphocytes, natural killer cells, tumor-associated macrophages (TAM) but rarely adipocytes. Tumor cells stimulate the infiltration of immune cells inside the TME [96]. Tumor-infiltrating immune cells and tumor cells interact with each other, then immune responses act to inhibit tumor growth. To this aim different stromal cells, immune cells, and CAFs closely interacting with cancer cells produce an inflammatory response, secrete growth factors and chemokines which unfortunately can promote tumor development, progression, and metastasis [97].

Over the last years, it has been proposed that the changes regarding tumor stroma metabolism are potent inducers of tumor growth [98]. In the phases of cancer induction and progression, normal fibroblasts, intimately connected to tumor cells undergo metabolic reprogramming altering phenotype. Cancer associated fibroblasts sustain the growth of neighboring epithelial cancer cells, causing in turn oxidative stress and senescence in adjacent CAFs [98]. In senescent CAFs, induction of autophagy and mitophagy potentiate a change on aerobic glycolysis producing biochemical molecules that drive oxidative phosphorylation anabolic growth in the tumor cells.

**Figure 3:**
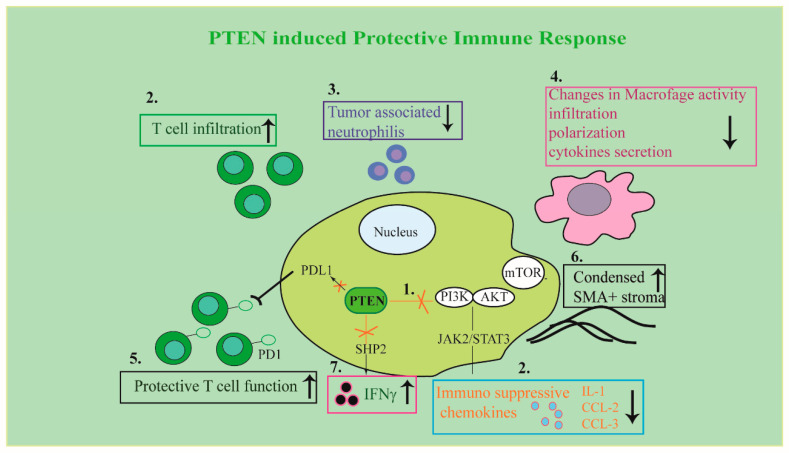
A schematic summary illustrating *PTEN* role in tumor/stroma interplay. See the text for details. (1) *PTEN* blocks oncogenic drivers of tumors including PI3K/AKT/mTOR and JAK2/STAT3. (2) *PTEN* reduces immunosuppressive cytokines, determining induction of T cells’ infiltration into tumor tissue. (3) *PTEN* reduces tumor-associated neutrophils (4) *PTEN* in macrophages reduces cytokines and M2 macrophage polarization in TME. (5) In tumor cells *PTEN* decreases PD-L1 expression which is responsible for T cell inactivation in the tumor microenvironment. (6) *PTEN* induces a condensed layer of SMA-positive stroma. (7) *PTEN* blocks PI3K-dependent activation of SHP 2 which acts as a negative regulator to inhibit IFN-γ.

Recent studies outline the onco-suppressor role of *PTEN* in the tumor microenvironment regulation, affecting metabolic reprogramming and autophagy. Indeed, specific genetic alterations including those of *PTEN* in cancer cells may affect the immune composition of the TME (see Figure 3 for a schematic representation), and such infiltrating immune cells may in turn act to inhibit or sustain cancer cells proliferation [99].

### 6.1. Regulation of TME Immune Response by PTEN

Mouse melanoma model carrying the frequent mutation of the BRAF oncogene (V600E) and *PTEN* deletion shows the repression of a protective immune response in the TME which was counteracted by an autophagy inducer, metformin, which determines tumor growth inhibition. Metformin causes the increase in the number of lung CD8-effector-memory T and CD4+ IL-10+ T cells and reduces metastasis in B16F10 melanoma cells transplanted mice [23]. These findings indicate that autophagy and *PTEN* inducers may contribute to treating melanoma carrying BRAF mutations, improving TME response. In drug-resistant ovarian cancer *PTEN* may cooperate with BECLIN 1, to block signaling inducing macrophage activity modification. Particularly, reduced expression of *PTEN* and BECLIN 1 was revealed in ovarian cancer tissues [100].

Furthermore, it is reported that *PTEN* deletion induces TME remodeling and is associated with immunosuppressive infrastructure in the TME. In the prostate of *PTEN*-knockout mice, much relaxed layers of smooth muscle actin (SMA)-positive malignant epithelial cells surrounding stroma are demonstrated, while a highly condensed layer of SMA-positive stroma, useful against the invasion of tumor cells into the adjacent stromal tissue, was detected in the prostate of wild-type mice (see Figure 3, point 6) [24].

Cross-talk between different signals during TME reprogramming is greatly influenced by genetic alterations of the *PTEN*/PI3K pathway. For instance, the stimulation of the Janus kinase (JAK) 2/Signal transducer and activator of transcription (STAT) 3 pathway as well as the secretion of such chemokines activating the infiltration of myeloid-derived suppressor cells contribute to *PTEN*-null prostate tumor growth and chemo-resistance (see Figure 3, points 1, 2) [101].

In glioblastoma (GBM) datasets, Chen and co-workers [25] evidence that poor outcomes and high stromal and immune signatures are related to *PTEN* loss and PI3K dysregulation but not with other pathway modifications. Deletion or mutation of *PTEN* are associated with higher infiltration of macrophage, a common cell type in GBM TME. The mechanism is dependent by *PTEN* deficiency promoting lysyl oxidase (LOX) expression, a potent macrophage chemoattractant in glioma cells. Lysyl oxidase, in turn, activates the β1 integrin dependent pathway in macrophages to promote their infiltration into GBM TME. This very elegant study evidences that these infiltrated macrophages secrete osteopontin (SPP1) promoting glioma cell survival and angiogenesis (see Figure 3, point 4).

In hepatocytes *PTEN* inactivation causes chronic damage that could remodel the hepatic microenvironment, recruiting inflammatory cells secreting cytokines and chemokines, driving hepatic cancer initiation and progression [9].

In vitro models simulating tumor under a nutrient depletion stress in the microenvironment show that miR-224 levels are inversely related to the expression of *PTEN*. Elevated miR-224 in tumor tissues compared with the adjacent normal tissues is correlated with tumor growth, decreased apoptosis, and autophagy [102].

All together these studies indicate that *PTEN* crucially contributes to immune-surveillance action in the complex interplay between tumor and stroma. This is confirmed in different cell contexts as the fibroblast-like synoviocytes increased concentrations of pro-inflammatory cytokines, chemokines, and VEGF expression are observed with *PTEN* inhibitor or *PTEN*-RNAi [103].

Thus, *PTEN* plays a critical role in TME, blocking a vicious cycle of positive feedback, responsible of aggressive local cell proliferation and metastatic spread to distant organs.

### 6.2. Cross-Talk Tumor–Stroma, PTEN Expression, and Function

In addition, *PTEN* loss-driven tumorigenesis is influenced by stromal cells inhibiting *PTEN* expression itself, in the tumor. [12]. In metastatic tumors exosomes containing anti-*PTEN* miRNAs secreted by stromal cells suppress *PTEN* expression in the tumor cells. *PTEN* reduction causes CCL2 chemokine secretion further increasing the outgrowth of metastatic tumor cells. Notably, stroma cells are effectors of *PTEN* loss-driven tumorigenesis through the secretion of factors potentiating the tumorigenic potential of cells. Loss of *PTEN* in macrophages, causes over-expression of cytokines that stimulate M2 macrophage to polarize. This is related to the increased infiltration of M2 macrophage, increased angiogenesis due to augmented vascular endothelial growth factor (VEGF) A and immune suppression in TME [104]. In high-grade serous ovarian carcinoma study, the number and/or localization of CD8+, CD45RO+, and CD68+ leucocytes, frequently associated with a patient’s overall survival [105], was not related with *PTEN* expression in the TME. This discrepancy could be related to a persistent low expression level of *PTEN* in these cases. Additional evidence supports the idea that the link between *PTEN* and the anti-tumor reprogramming of the stroma is mediated by the interferon signaling pathway. It was published that lung adenocarcinoma cells express low levels of *PTEN* and are interferon (IFN) γ insensitive and restoring *PTEN* expression reverses those effects [26]. The signal transduction of IFN γ is related with glycogen synthase kinase (GSK)-3β upregulation and autophagic induction. Conversely, abnormal PI3K stimulation and reduction of *PTEN* is accompanied by GSK-3β inactivation and the activation of Src homology 2-containing phosphatase (SHP) 2 which act as negative regulator to inhibit IFN-γ (see Figure 3, point 7).

## 7. Conclusions

In conclusion *PTEN* is a powerful tumor suppressor and its loss of function is often detected in heritable and sporadic cancers. Subtle changes in *PTEN* levels drive cancer predisposition as well as tumor progression, emphasizing the essential role of *PTEN* regulated mechanisms in cellular homeostasis and tumorigenesis. In recent years in vitro and in vivo studies have delineated a new position of *PTEN* function within the cell, such as key controller of metabolic states, also through the activation of autophagic phenotype and anti-Warburg effect, for a tumor suppressor action. Given the fact that energy request tightly determines cell proliferation and survival of cancer cells, the elucidation of the mechanisms governing *PTEN* expression and function acquires important therapeutic implication for an extensive selection of human cancers and inheritable syndromes associated with abnormal signals regulated by *PTEN*. Notably, *PTEN* contributes to the regulation of reciprocal interplay between cancer cells and the TME, exerting in such a way a “tumor-suppressive/immune-protective effect”. For instance, *PTEN* genetic alterations in several type of cancers influences the immune composition of the TME and infiltrating immune cells function to modulate the growth of tumor cells. These evidence strongly highlights the potential clinical benefit of a therapeutic action targeting *PTEN* which functions inside the interaction between cancer cells and immune cells. Several other questions persist to be elucidated and additional investigations could identify the best strategies for the restoration of *PTEN* function in cancer prevention and treatment.

## Figures and Tables

**Table 1:** List of *PTEN* alteration, observable effects, and experimental models.

Type of Cancer	PTEN Alteration	Observable Effects	Models	References
**Thyroid**	PTEN loss	Down-regulation of Krebs cycle and OXPHOS gene expression, defective mitochondria, reduced respiration and compensatory glycolysis.	PTEN-deficient mouse model	[18]
**Liver**	PTEN Knockdown	Increased oxidative stress levels, increase of reactive oxygen species (ROS), elevated genomic damage	PTEN deficient mice maintained with a high fat diet	[19]
**Glioblastoma**	Ectopic expression of WT PTEN and mutants	Effects on autophagic flux and lysosomal mass.	U87MG human glioma cells	[20]
**Hepatocellular carcinoma**	PTEN loss	Suppression of autophagy at the formation and maturation steps of autophagosomes	Hepatocyte-specific PTEN-deficient mice	[21]
**Breast cancer**	Up-regulation of PTEN	Induction of autophagy and reduction of breast cancer cell growth	Hormone dependent breast cancer cells	[15]
**Melanoma**	PTEN loss	Induction of immunosuppressive cytokines, reduction of T-cells infiltration in tumor tissue, inhibition of autophagy and T cell–mediated cell death	PTEN- deficient melanoma mouse models	[22]
**Melanoma**	Cre-inactivated allele of PTEN	Repression of a protective immune response in the tumor microenvironment, increase in tumor growth and metastasis	BRAF V600E/PTEN loss murine melanoma models	[23]
**Prostate**	PTEN Knockdown	Induction of tumor microenvironment remodeling, associated with immunosuppressive infrastructure	PTEN conditional knockout mice	[24]
**Glioblastoma**	PTEN Knockdown or PTEN mutation	Infiltration of macrophage that secrete factors promoting glioma cell survival and angiogenesis.	PTEN null glioma mouse models	[25]
**Liver**	PTEN Knockdown	Spontaneous development and progression of liver tumors from progenitor cells. Induction of hepatic microenvironment remodeling.	PTEN-null liver mouse models	[9]
**Lung**	PTEN silencing targeting human PTEN	Reduction of IFN—induced inflammatory response, cell growth inhibition, and cytotoxicity	A549 and PC14PE6/AS2 human lung adenocarcinoma cells	[26]
